# Biomimetic, mussel-inspired surface modification of 3D-printed biodegradable polylactic acid scaffolds with nano-hydroxyapatite for bone tissue engineering

**DOI:** 10.3389/fbioe.2022.989729

**Published:** 2022-09-08

**Authors:** Minghan Chi, Na Li, Junkui Cui, Sabrina Karlin, Nadja Rohr, Neha Sharma, Florian M. Thieringer

**Affiliations:** ^1^ Medical Additive Manufacturing Research Group (Swiss MAM), Department of Biomedical Engineering, University of Basel, Allschwil, Switzerland; ^2^ Department of Earth and Environmental Studies, Montclair State University, Montclair, NJ, United States; ^3^ Biomaterials and Technology, Department of Research, University Center for Dental Medicine Basel UZB, University of Basel, Basel, Switzerland; ^4^ Biomaterials and Technology, Department of Reconstructive Dentistry, University Center for Dental Medicine Basel UZB, University of Basel, Basel, Switzerland; ^5^ Oral and Cranio-Maxillofacial Surgery, University Hospital Basel, Basel, Switzerland

**Keywords:** bioinspired, three-dimensional printing, hydroxyapaite, surface modication, nanocomposites

## Abstract

Polylactic acid (PLA) has been widely used as filaments for material extrusion additive manufacturing (AM) to develop patient-specific scaffolds in bone tissue engineering. Hydroxyapatite (HA), a major component of natural bone, has been extensively recognized as an osteoconductive biomolecule. Here, inspired by the mussel-adhesive phenomenon, in this study, polydopamine (PDA) coating was applied to the surface of 3D printed PLA scaffolds (PLA@PDA), acting as a versatile adhesive platform for immobilizing HA nanoparticles (nHA). Comprehensive analyses were performed to understand the physicochemical properties of the 3D-printed PLA scaffold functionalized with nHA and PDA for their potent clinical application as a bone regenerative substitute. Scanning electron microscopy (SEM) and element dispersive X-ray (EDX) confirmed a successful loading of nHA particles on the surface of PLA@PDA after 3 and 7 days of coating (PLA@PDA-HA3 and PLA@PDA-HA7), while the surface micromorphology and porosity remain unchanged after surface modification. The thermogravimetric analysis (TGA) showed that 7.7 % and 12.3% mass ratio of nHA were loaded on the PLA scaffold surface, respectively. The wettability test indicated that the hydrophilicity of nHA-coated scaffolds was greatly enhanced, while the mechanical properties remained uncompromised. The 3D laser scanning confocal microscope (3DLS) images revealed that the surface roughness was significantly increased, reaching Sa (arithmetic mean height) of 0.402 μm in PLA@PDA-HA7. Twenty-eight days of *in-vitro* degradation results showed that the introduction of nHA to the PLA surface enhances its degradation properties, as evidenced by the SEM images and weight loss test. Furthermore, a sustainable release of Ca^2+^ from PLA@PDA-HA3 and PLA@PDA-HA7 was recorded, during the degradation process. In contrast, the released hydroxyl group of nHA tends to neutralize the local acidic environments, which was more conducive to osteoblastic differentiation and extracellular mineralization. Taken together, this facile surface modification provides 3D printed PLA scaffolds with effective bone regenerative properties by depositing Ca^2+^ contents, improving surface hydrophilicity, and enhancing the *in-vitro* degradation rate.

## Introduction

Critical-sized bone defects are severe consequences of traumatic injury, infection, congenital defects, or surgical resection, which require clinical interventions to achieve functional restoration and complete healing (Zhang et al., 2019). Although bone autografts are the gold standard for bone reconstructive surgeries, their application is limited due to additional pain, potential infection, and donor-site morbidity (Blokhuis and Arts, 2011). Thus field of bone tissue engineering thus aims to design and develop materials that outperform bone allografts and autografts (Roseti et al., 2017). Despite many conventional approaches that have been developed for fabricating biomimetic porous scaffolds (Chen et al., 2018; Grenier et al., 2019; Sola et al., 2019), challenges remain to optimize the shape and porosity of patient-specific customized scaffolds. Nowadays, with the advancement of materials and medical technology, additive manufacturing (AM), also known as three-dimensional (3D) printing, has attracted significant attention to producing predictable bone regenerative scaffolds with customized shapes and structures (Brien, 2011; Madrid et al., 2019).

Polylactic acid (PLA), one of the most intriguing polymeric materials, has recently received much attention in the food and medical fields due to its excellent biodegradability, biocompatibility, bioresorbability, and ductility (Senatov et al., 2016; Tyler et al., 2016). However, PLA also has the disadvantages of being bioinert, hydrophobic, low fracture toughness, and lacking osteoconduction and cell-scaffold interactions, limiting its potential clinical application (Ma et al., 2007; Mondal et al., 2020; Zaaba and Jaafar, 2020). To tackle such drawbacks, nanosized hydroxyapatite (nHA) has been widely introduced to the bone regenerative scaffolds as an additive composite or coating material to act as a cell recognition site and promote bone regeneration (Dutta et al., 2015; Ramesh et al., 2018). Various conventional fabricating methods, such as solvent casting, mechanical milling, and electrospinning, have been applied to produce PLA/nHA composite scaffolds for bone regeneration (Novotna et al., 2014; Trifol et al., 2019). However, the presence of residual organic solvents (e.g., chloroform and dichloromethane) resulted in harmful effects on cells and tissues (Chakraborty et al., 2018; Gayer et al., 2019). More importantly, the surface of scaffolds was not ideally functionalized since most of the nHA particles are entrapped in the PLA matrix, resulting in limited hydrophilicity, cell recruitment, and osteo-stimulating effects (Qiang et al., 2018; Liu et al., 2020).

In recent years, the mussel-inspired, biomimetic polydopamine (PDA) coating has received increasing attention as a universal bio-adhesive coating due to its unique adhesion ability (Lee et al., 2007; Grewal and Yabu, 2020; Grewal et al., 2021). With material-independent surface chemistry and deposition strategy, the PDA coating layer can be quickly formed at different interfaces (e.g., oil-water and air-water interfaces) by oxidation and self-polymerization of dopamine (DA) (Ryu et al., 2010) and thus can be expected promising applications in multidisciplinary fields such as energy, environmental, electrocatalysis, and biomedicine (Liu et al., 2014; Abe et al., 2020; Abe and Yabu, 2021; Yabu et al., 2022). The as-synthesized PDA layer further serves as a versatile platform for the immobilization of bionic molecules (e.g., bioactive ions and ceramics) (Shanmugam et al., 2019; Park et al., 2021; Shuai et al., 2021).

Herein, a facile approach to fabricating osteoconductive and osteoinductive nanocomposite scaffolds using material extrusion-based (commonly known as Fused Filament Fabrication, (FFF)) AM technology with surface modification strategy is presented in the present work. This study aims to comprehensively analyze additively manufactured PLA scaffolds functionalized by PDA and nHA, from surface structure to *in-vitro* degradation properties. The objectives of this study were to develop 3D printed porous scaffolds with enhanced osteogenicity by introducing PDA and nHA to their surfaces and to systematically characterize and evaluate the scaffolds’ physicochemical properties, including surface hydrophilicity, roughness, mechanical behavior, and *in-vitro* biodegradability. The hypotheses were that *1*) the PDA and nHA coating result in increased hydrophilic surfaces of PLA scaffolds and *2*) the nHA and PDA coating enhances the *in-vitro* hydrolysis process of PLA scaffolds, and *3*) the mechanical properties were not compromised.

## Materials and methods

### Materials

Dopamine hydrochloride ((HO)_2_C_6_H_3_CH_2_CH_2_NH_2_·HCl), tris-base (NH_2_C(CH_2_OH)_3_), hydrochloric acid (HCl, 98%), and phosphate-buffered saline (PBS) at pH = 7.4 were purchased from Sigma-Aldrich (Merck KGaA, Darmstadt, Germany). Material extrusion 3D printing PLA filaments were purchased from 3DJake GmbH (Paldau, Austria). Nano hydroxyapatite (nHA) powders (≥97%, <200 nm particle size-BET) were obtained from Shanghai Aladdin Biochemical Technology (Shanghai, China). Ultrapure water (>18.2 MΩ∙cm) was provided using a Milli-Q ^®^ EQ water purification system (Basel, Switzerland).

### Fabrication of material extrusion 3D printed PLA scaffolds

3D scaffolds (10 mm × 10 mm × 3 mm) were designed using a computer-aided design modeling software (3ds Max, v. 2022, Autodesk Inc., San Francisco, CA, United States) and exported in a standard tessellation language (STL) file format. The printing path of the scaffolds was then generated using the 3D printer’s compatible slicing software (MakerBot Print software, v. 3.10.1, NY, United States) and saved in a g-code file. Specifically, the parameters of g-code were set at a printing temperature of 215°C, layer height of 0.2 mm, infill density of 90%, infill rotation angle of 90°, and travel speed of 150 mm/s. Subsequently, the g-code was imported to the material extrusion-based 3D printer (Makerbot Replicator +, Makerbot Industries, NY, United States) with a nozzle of 400 μm and printed using a diameter of 1.75 mm PLA filament. After production, all scaffolds were stored at room temperature in a desiccator before further surface modification and characterization.

### Surface modification of material extrusion 3D printed PLA scaffolds

**FIGURE 1:**
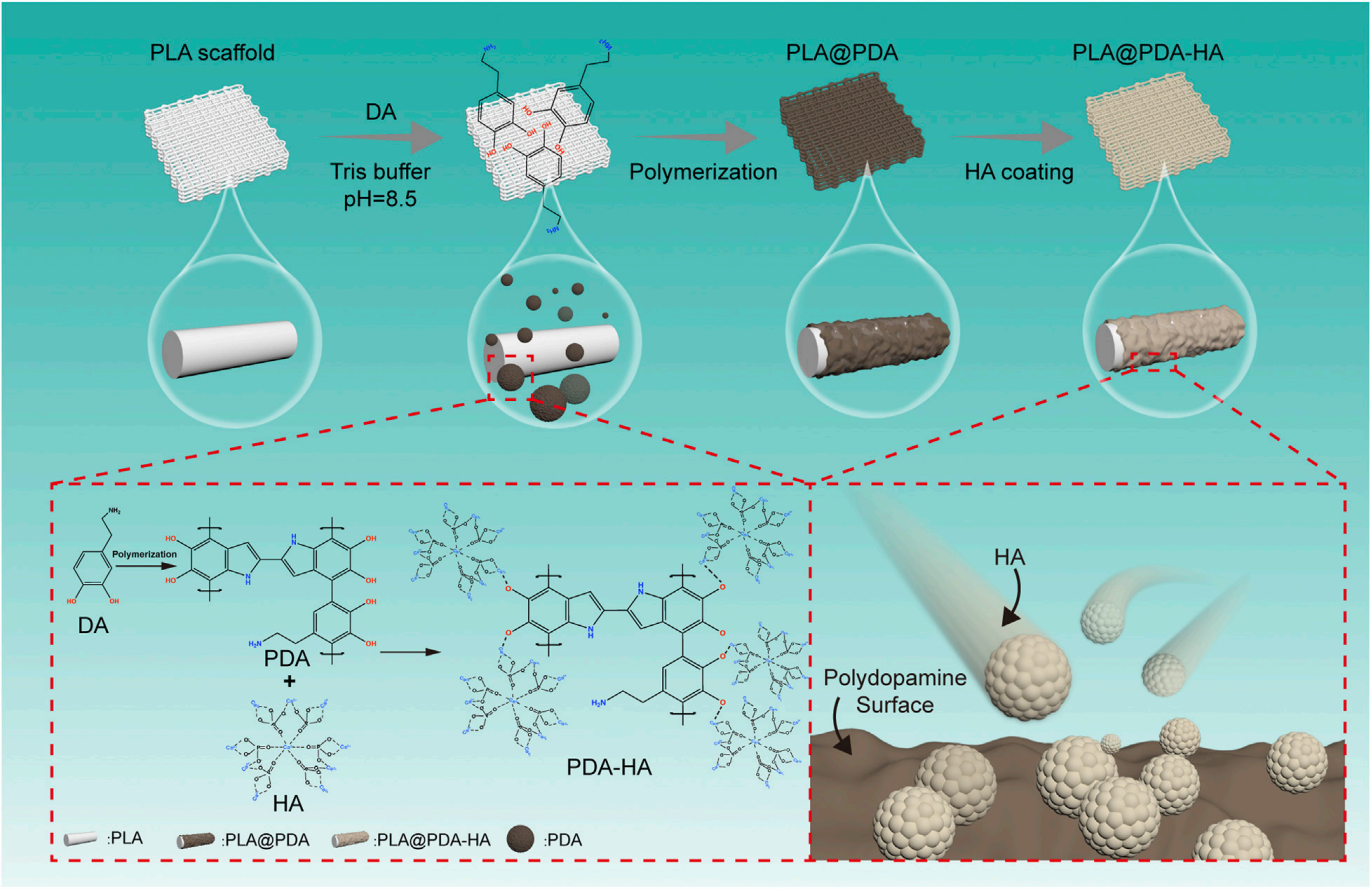
Schematic illustration of surface modification process of three-dimensional (3D)-printed PLA scaffolds. The PLA scaffold was fabricated with a rectangular porous structure using material extrusion-based 3D printing technology. Afterward, polydopamine was homogeneously coated onto the surface by immersing PLA scaffolds into the dopamine solution (tris buffer, pH = 8.5) and simultaneously stirring for 24 h, during which PDA was developed by self-polymerization of DA particles. Furthermore, nHA particles were successfully immobilized onto the surface of as-synthesized PDA coatings *via* catechol functional groups. Finally, the nHA functionalized PLA scaffolds were obtained.

The overview of the surface modification process was depicted in the schematic illustration (Figure 1). The PLA scaffolds were fabricated using material extrusion-based 3D printing technology. Subsequently, simple and direct coating methods were applied to obtain PDA and nHA functionalized PLA 3D scaffolds. The surface functionalization of material extrusion 3D printed PLA scaffolds with PDA was carried out as illustrated by Lee et al. (Lee et al., 2020). Briefly, the spontaneous formation of a thin adherent PDA film on PLA scaffolds was achieved *via* direct immersion into a dopamine solution (2 mg/ml in 10 mM Tris buffer, pH 8.5) under a 25 rpm shaker (Agilent Technologies, CA, United States) at room temperature (25°C) for 24 h. The solution was continuously stirred during the coating process to avoid the aggregation of self-polymerizing PDA nanoparticles formed in the solution. The resultant PLA scaffolds, denoted as PLA@PDA, were rinsed with deionized water three times to remove the residual solvent and dried at 60°C in a conventional oven. Immobilization of HA nanoparticles was achieved by immersing PLA@PDA scaffolds into nHA solution (50 mg/ml) for 3 and 7 days. Finally, the scaffolds were washed with deionized water three times, dried overnight at 60°C, and hereafter referred to as PLA@PDA-HA3 and PLA@PDA-HA7. Native 3D printed PLA scaffolds without surface modification served as the control group (henceforth referred to as PLA).

### Microstructure characterization

The micromorphologies and chemical composition of scaffolds were investigated using scanning electron microscopy (SEM, Phillips XL30, Eindhoven, Netherlands) equipped with energy-dispersive X-ray spectroscopy (EDX). The scaffolds were sputtered with gold and observed at an accelerating voltage of 10 kV at different magnifications. EDX analysis was conducted using an accelerating voltage of 20 kV. The porosity of 3D-printed PLA scaffolds was obtained using the following equation:

Porosity(%)=1−(ρscaffolds/ρpla)×100% (1)

The ρ scaffolds was defined as the ratio of weight and volume of the scaffolds, while the ρ pla was 1.25 g/cm3 according to previously reported (Van der Walt et al., 2019).

The phase composition of scaffolds was studied using X-ray diffraction (XRD). The XRD patterns were recorded in the 2θ range 5°–40°, with a step size of 0.02°. Specifically, the XRD test specimens were printed in a cylindrical shape (D: 10 mm; H: 1 mm), followed by surface modification procedures. The functional groups of scaffolds with or without surface modification were identified using Fourier transform infrared spectroscopy with attenuated accessory (FTIR-ATR) in the range of 500–4000 cm^−1^. The surface roughness of each specimen was visualized and studied using Keyence Laser Microscope (VK-X3000 series, Keyence Corporation, Osaka, Japan).

### Water absorption ability and surface wettability

To determine the water absorption ability of scaffolds, using a contact angle system, a drop shape analyzer (DSA 100, Krüss, Hamburg, Germany) tested the surface wettability (hydrophilic or hydrophobic) of the scaffolds. The samples were placed on a microscope glass slide, and three 2 μl ultrapure water droplets were applied to each sample at room temperature. Moreover, the water uptake ability of 3D printed porous samples was measured. Briefly, the dried scaffolds were first weighed and then immersed in a 5 ml PBS solution for 10, 30, and 60 s. The samples were weighed using filter paper after removing the residual water on the surface at each predetermined time point. The water absorption ability or water uptake (%) of the scaffolds was calculated according to the equation:

Water Uptake(%)=[W(s)-W(i)]/W(i)×100%

Where W(s) is the weight of the scaffold after immersion, and W(i) is the initial dry mass. All tests were performed in three replicates.

### Mechanical tests

To identify the mechanical properties of scaffolds, tensile strength and compressive strength were studied using a universal testing machine (Z020, Zwick/Roell, Ulm, Germany). Briefly, the 3D model of a tensile bar was designed and created based on the International Organization for Standardization (ISO) 527-1 (2012) standard (detailed dimension and print patterns refer to Supplementary Figure S1) (Iof, 2012). All specimens used in tensile tests were sliced and printed under the previously described condition. The grip distance was 25.4 mm, and the speed was 5 mm/min. The compressive tests were performed on the fabricated scaffold samples (10 mm × 10 mm × 3 mm) with and without surface modification at a crosshead speed of 1 mm/min. All mechanical tests were performed in three replicates, and the mean values of each group were reported.

### Thermogravimetric analysis

To investigate the amount of HA nanoparticles immobilized on PLA scaffolds, thermogravimetric analysis (TGA) was performed (TGA 5500, TA Instruments, New Castle, DE, United States). The samples were first cut into small pieces (3 mg) and placed in aluminum pans. Samples were then heated to 800°C at a ramp rate of 10°C min^−1^ under nitrogen flow. The residues were considered the inorganic contents. The experiments were conducted in three replicates.

### *In-vitro* biodegradable properties

*In-vitro* degradation ratios of the study groups’ samples were measured *via* the mass loss method. In short, the dried scaffolds were initially weighed and immersed in PBS solution and kept at 37°C for 28 days. At each predetermined time point, the samples were removed and dried overnight, and the dry weight was recorded and compared with the initial weight to determine the degradation rate. At the end of each time point, the pH variation of PBS solution was detected utilizing a pH meter (AE150, ThermoFisher Scientific, MA, United States). At 14 and 28 days, the degradation micro-morphologies of samples were observed by SEM. Furthermore, calcium ion release rates of PLA@PDA-HA3 and PLA@PDA-HA7 scaffolds were studied. The release of calcium ions was determined at predetermined time intervals of 7, 14, 21, and 28 days. The immersion solution was collected and renewed with fresh PBS at each time point. The collected solutions were stored at 4°C until measurements. Finally, each collected supernatant’s calcium ion concentration (ppm) was investigated *via* inductively coupled plasma mass spectrometry (ICP-MS, ICAP Q, ThermoFisher Scientific, MA, United States).

### Statistical analysis

The data were expressed as means ± standard deviation (SD) of independent replicates. The results of the experiments were statistically analyzed using a one-way analysis of variances (ANOVA) using the GraphPad Prism software package (GraphPad, San Diego, CA, United States). Significant differences among mean values, where applicable, were determined using ANOVA and by Tukey’s *post hoc* test for multiple comparisons. The level of significance was set to α = 0.05.

## Results

### Fabrication and characterization of nHA-coated scaffolds

**FIGURE 2:**
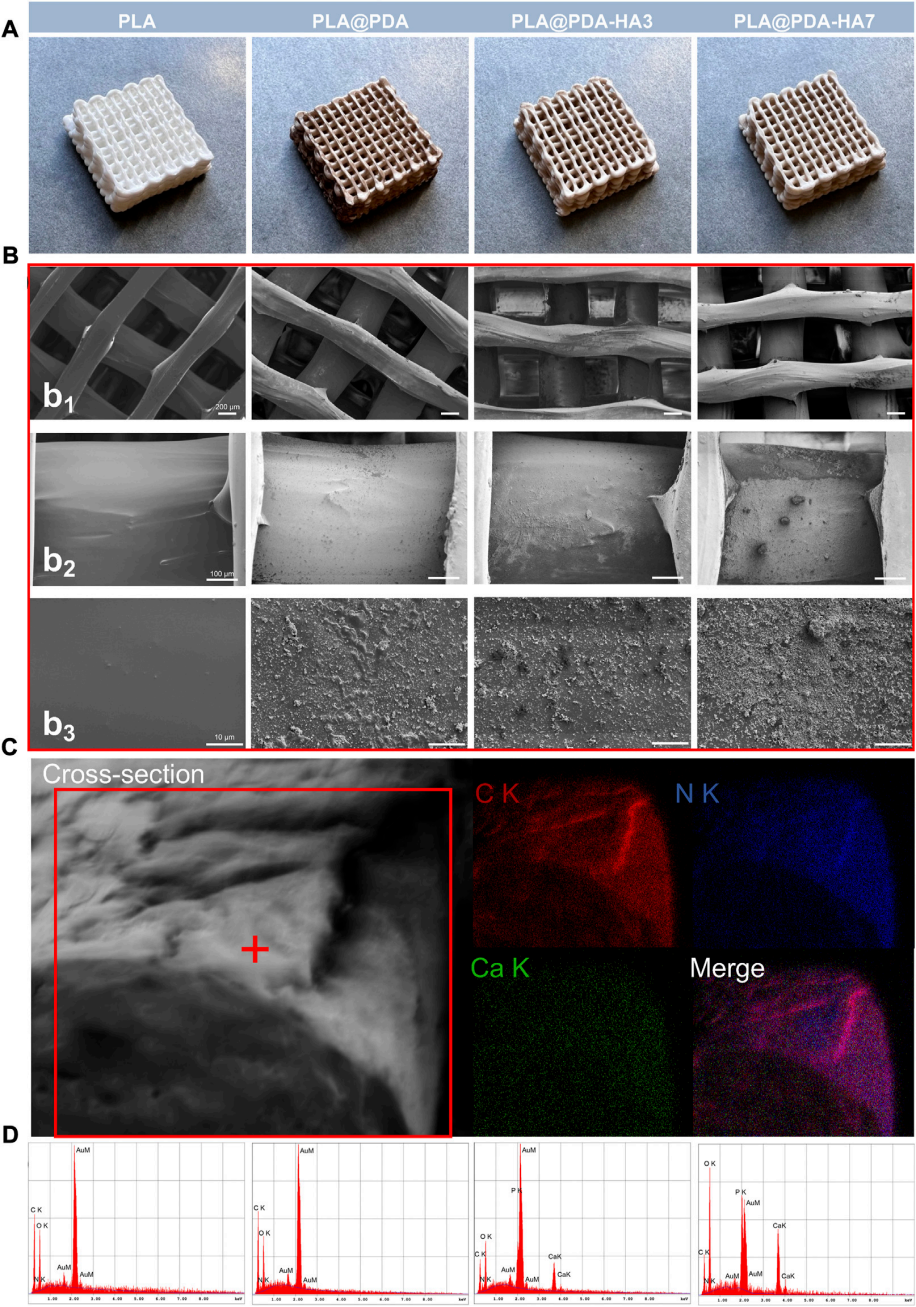
**(A)** Representative optical images of 3D printed PLA, PLA@PDA, PLA@PDA-HA3, and PLA@PDA-HA7 scaffolds; **(B)** Corresponding SEM images of surface topography of the fabricated scaffold within each group. Scale bar: Figure 2b_1_ = 200 μm, Figure 2b_2_ = 100 μm, Figure 2b_3_ = 10 μm; **(C)** Cross-sectional micromorphology of PLA@PDA-HA7 specimen and the corresponding EDX mapping. (Red: Carbon; Blue: Nitrogen; Green: Calcium). **(D)** Corresponding EDX analysis of each scaffold.

The fabricated scaffolds’ representative photographs and surface micro-morphology are shown in Figures 2A,B, respectively. Representative macrostructure digital photographs (Figure 2A) revealed that the overall architecture of 3D-printed PLA (native and functionalized nanocomposite) scaffolds was with a well-designed rectangular subunit depicting lattice periodicity, and no fabrication defects associated with the spreading of the coatings on polymer were observed. In contrast to the native 3D-printed PLA scaffolds, the PDA functionalized PLA scaffolds turned from white to dark brown, indicating the successful oxidative self-polymerization of DA with the production of eumelanin on the surface of PLA scaffolds. Similarly, after coating with the HA nanoparticles, the PDA functionalized PLA scaffolds exhibited a creamy-white color, indirectly demonstrating the successful loading of nHA. SEM images at different magnifications further confirm the abovementioned findings. The lower magnification SEM images (Figure 2b_1_) show the struts and pores’ morphology of the fabricated scaffolds. The struts of scaffolds with a diameter of ∼600 μm were printed neatly, and a ∼400 μm interconnected pores were shown, and they remained unchanged with the surface modification process.

Moreover, highly interconnected porosity was also observed, following the pre-modeled design. High magnification SEM images (Figures 2b_2_ and 2b_3_) clearly showed the fabricated scaffolds’ different surface morphology and topography. The PLA scaffolds exhibited smooth and uniform surfaces, while lumps of PDA deposits were coated homogeneously all over the surfaces, indicating PDA was successfully deposited to the surface. For the PLA@PDA-HA3 scaffolds, there were some relatively small amounts of nHA distributed on the surface. However, it can be observed that apart from the area coated with nHA, a large portion of PLA surfaces was not covered, leaving it exposed. In contrast, with the extension of immersion time to 7 days, thicker and more visible nHA coatings were formed evenly on the surface of PLA@PDA-HA7. Additionally, the surfaces of PLA@PDA-HA7 were covered by different sizes of nHA aggregates, suggesting that 7 days of immobilization were sufficient to achieve high loading of HA nanoparticles.

Figure 2C shows the cross-sectional micromorphology of the PLA@PDA-HA7 scaffold. The surface of the strut appeared rougher due to the coating of nHA and PDA. The corresponding EDX-mapping analysis showed a homogeneous element distribution on the fracture surface, with strong signals of C (red) and N (blue) signals on the interior part of the strut. In contrast, relatively weak Ca (green) signals were concentrated on the surface area due to the nHA coating.

EDX was carried out to verify these results further and determine the chemical composition of different scaffold surfaces (Figure 2D and Table 1). As shown in Table 1, the nitrogen (N) content (wt%) increased from 13.6 to 18.4 wt% in PLA@PDA scaffolds indicating successful coating of PDA layers on the PLA scaffold surface. Elements Ca and P were distributed on the surfaces after immersing scaffolds in nHA solution for 3 and 7 days, with a concomitant increase. Moreover, the resulting Ca/P ratios of PLA@PDA-HA3 and PLA@PDA-HA7 were 1.69 and 1.54, respectively (Table 1), which is close to the theoretical Ca/P ratio of HA 1.67. Additionally, the porosity rate of 3D printed PLA scaffolds showed a minor oscillation rate (Supplementary Table S1), with 36% ± 0.005 (*p* < 0.05), which was within the range of cancellous bone (Bose et al., 2012).

**TABLE 1:** Elements obtained with EDX on the surface of each specimen in Wt%.

	*Element (wt%)*					
Group	C K	O K	N K	Ca K	P K	Total
PLA	40.0	46.4	13.6	—	—	100
PLA@PDA	38.6	43.0	18.4	—	—	100
PLA@PDA-HA3	26.0	33.0	10.2	21.2	9.6	100
PLA@PDA-HA7	9.8	33.5	4.6	34.7	17.4	100

### Chemical and phase composition of scaffolds

**FIGURE 3:**
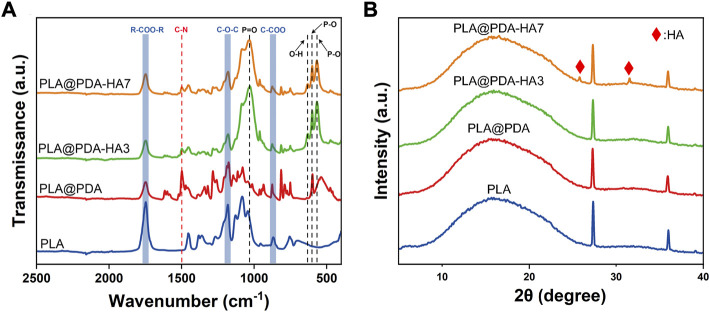
FTIR-ATR spectrum **(A)** and XRD pattern **(B)** of PLA, PLA@PDA, PLA@PDA-HA3, and PLA@PDA-HA7 scaffolds.

The chemical composition of the specimens was studied using FTIR-ATR analysis. As shown in Figure 3A, all four groups showed typical peaks at 1750 cm^−1^, 1181 cm^−1^, and 869 cm^−1^, which were assigned to the carboxyl group stretch vibration (V_R-COO-R_), and ether group stretch vibration (V_C-O-C_), reflecting the carbon backbone of the PLA. For the PLA@PDA sample, a new peak at 1498 cm^−1^ corresponding to the C-N group stretch vibration appeared. Additionally, the intensity of this peak decreased significantly after introducing nHA to the surface. In PLA@PDA-HA3 and PLA@PDA-HA7 scaffold samples, multiple new peaks at 1040 cm^−1^ (V_P=O_), 600 cm^−1^, 566 cm^−1^ (V_P-O_), and 631 cm^−1^ (OH^−^) were found, indicating the presence of PO_4_^3-^ and thus confirming the successful immobilization of HA.

The XRD tests were also carried out to confirm the phase composition of the 3D printed scaffold samples. Figure 3B shows the diffraction peaks of PLA, PLA@PDA, PLA@PDA-HA3, and PLA@PDA-HA7 scaffolds. For the PLA scaffolds, it offers a typical broad peak at 2θ ≈ 17°, which was the characteristic peak of PLA, and two sharp peaks at 2θ ≈ 27.8° and 35.4°, respectively, corresponding to the (200) and (110) plane of the orthorhombic crystal. Compared with PLA scaffolds, no new Bragg peaks were detected in the PLA@PDA scaffold samples due to the amorphous structure of PDA. After immobilizing nHA on the surface of PLA@PDA for 3 days, no new typical diffraction peaks of crystalline HA were observed. When nHA coating was extended to 7 days, two characteristic diffraction peaks at around 25.9° and 31.8° arose, corresponding to the (002) and (211) crystal planes of HA nanoparticles.

### Surface roughness and 3D topography of scaffolds

**FIGURE 4:**
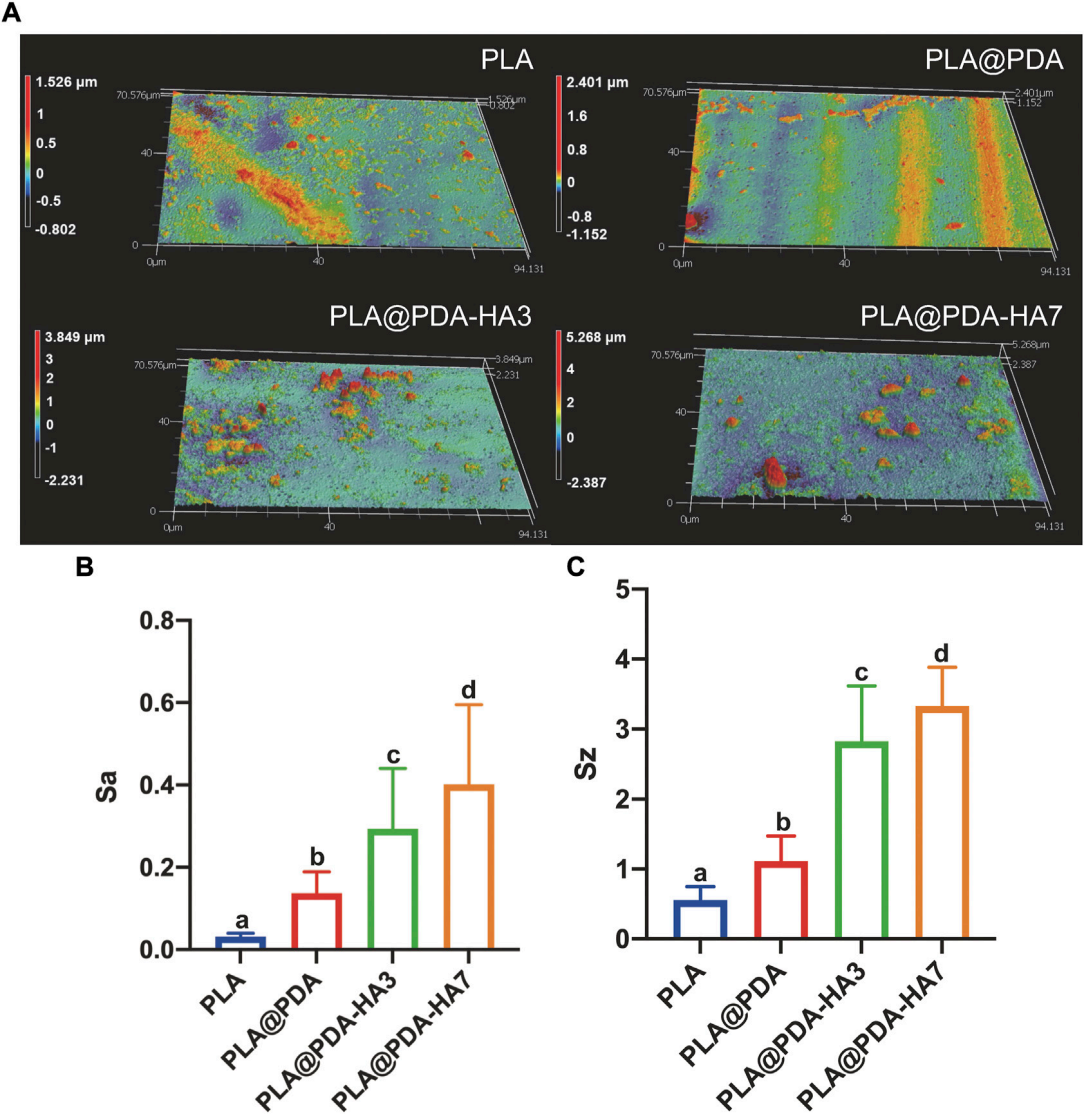
Surface roughness evaluation of PLA, PLA@PDA, PLA@PDA-HA3, and PLA@PDA-HA7 scaffolds. **(A)** Representative 3D surface topography of each scaffold. **(B)** Corresponding Sa (arithmetic mean height) and **(C)** Sz (maximum height). Dissimilar letters indicate statistical differences between groups (*n* = 3, *p <* 0.05).

Surface 3D topography and roughness of different scaffold samples (scanning area of 94.1 × 70.6 μm rectangle) are illustrated in Figure 4. Unlike PLA and PLA@PDA scaffolds, apparent peaks and valleys were noticed in nHA-coated samples representing HA nanoparticles and aggregates (Figure 4A). Figures 4B,C illustrates the surface roughness variation trends of Sa (arithmetical mean height) and Sz (maximum height). Following SEM images, Sa and Sz increase with the extension of nHA coating time. Precisely, compared with the Sa of native PLA scaffolds (ca. 0.03 µm), it can be calculated that the Sa of PLA@PDA, PLA@PDA-HA3, and PLA@PDA-HA7 increased to 0.14, 0.29, and 0.40 µm respectively. Similarly, the Sz value increased correspondingly and reached the highest level of 3.33 µm in PLA@PDA-HA7 scaffold samples.

### Water contact angle and wettability tests

**FIGURE 5:**
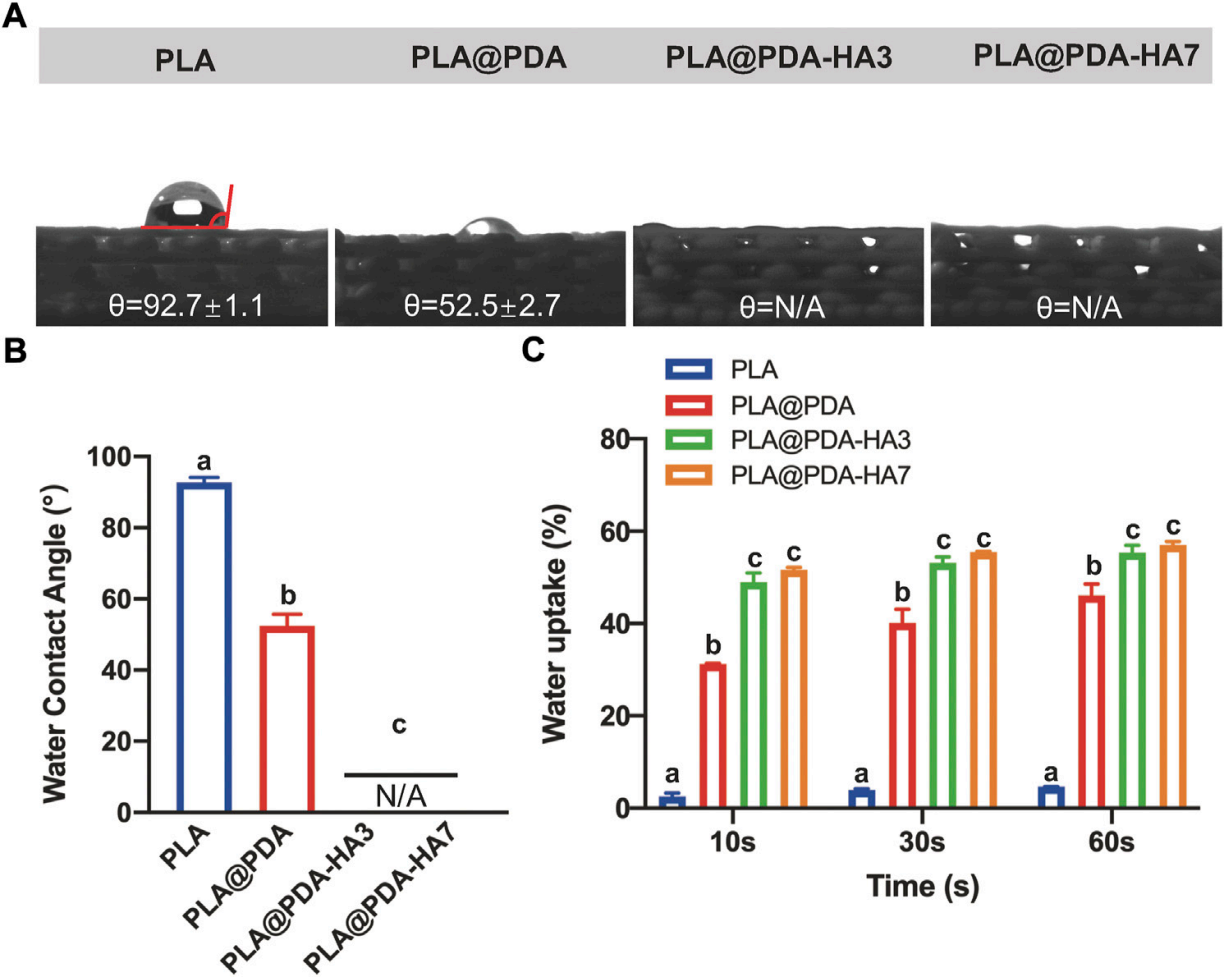
Hydrophilicity assay of PLA, PLA@PDA, PLA@PDA-HA3, and PLA@PDA-HA7 scaffolds. **(A)** Representative photographs of water droplets on the surface of each scaffold. **(B)** The water contact angle within each group. **(C)** Wettability analysis of each scaffold immersed in PBS for 10, 30, and 60 s. Dissimilar letters indicate statistically significant differences between groups (*n* = 6, *p <* 0.05).

Figures 5A,B shows the water contact angle test (WCA) result. Compared with PLA scaffolds, the hydrophilicity of the PLA@PDA scaffold surface improved significantly, evidenced by a decrease in WCA from 92.7° to 52.5°. Interestingly, after functionalizing the PLA scaffold with nHA, the surface became completely hydrophilic and absorbed the water drops as soon as they fell onto the surface. Consequently, the WCA of PLA@PDA-HA3 and PLA@PDA-HA7 could be either 0° or undetectable. Figure 5C shows the water uptake rate of each scaffold during the time of 60 s. PLA scaffold showed an inferior water uptake ability, only achieving 4.7% after 60 s immersion. In contrast, the surface modification process enabled the scaffolds to absorb higher water. The PLA@PDA scaffold absorbed water more rapidly and significantly (*p* < 0.05), reaching 31.2% at 10 s and the maximum water absorption rose to 46.1% after 60 s. This trend became more evident with nHA coating, with 55.4% and 57% water absorption rates for PLA@PDA-HA3 and PLA@PDA-HA7 scaffold samples. However, no statistical significance was found between these two groups (*p* = 0.950). Additionally, during the water uptake tests, it was found that the surface-modified scaffolds sank immediately after placing them in PBS solution, whereas the PLA scaffold was floating on the solution during the 60 s experiment duration (*see* Supplementary Figure S2).

### Mechanical behaviors of scaffolds

**FIGURE 6:**
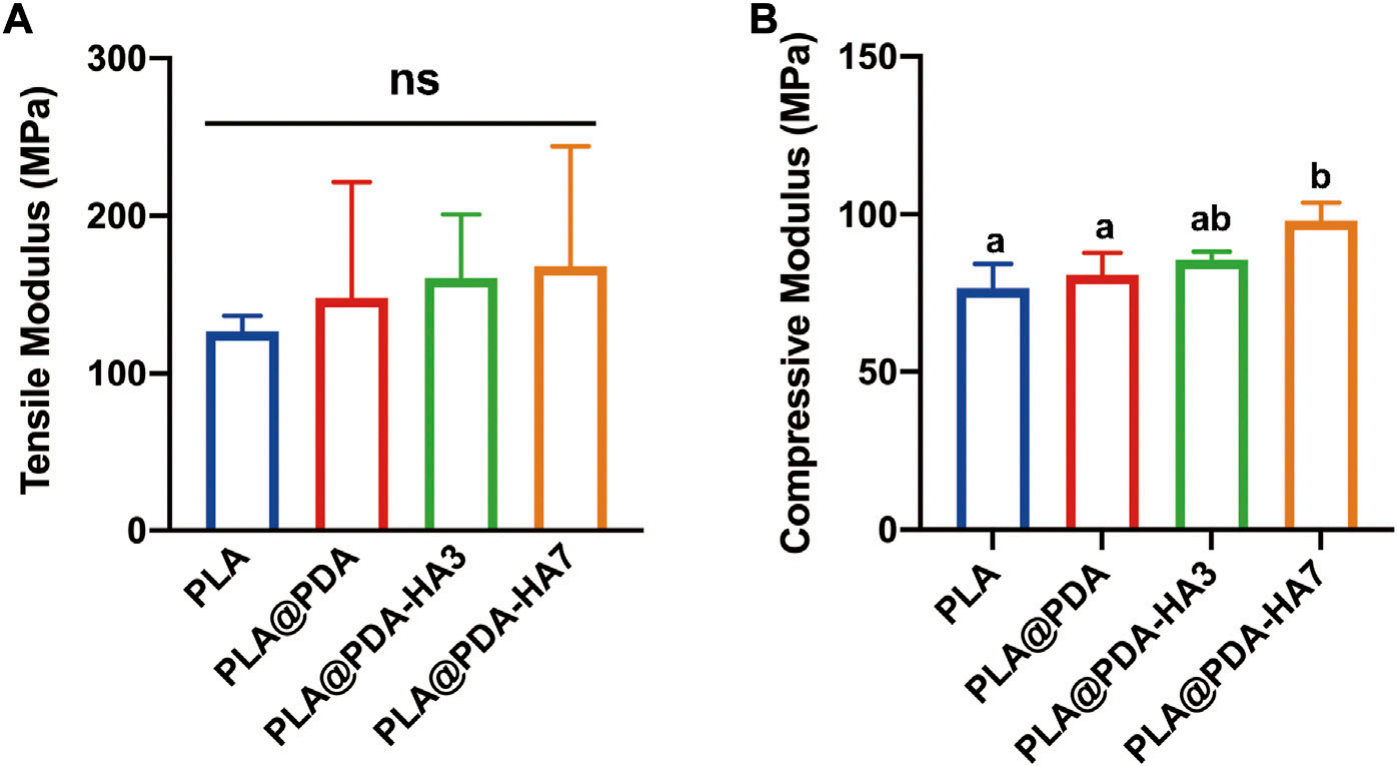
Mechanical properties of PLA, PLA@PDA, PLA@PDA-HA3, and PLA@PDA-HA7 scaffolds. **(A)** Tensile modulus and **(B)** compressive modulus of each specimen. Dissimilar letters indicate statistical differences between groups (*n* = 3, *p <* 0.05).

The mechanical properties of each scaffold were determined *via* tensile and compressive strength tests, and the corresponding young’s modulus was calculated from the stress-strain curves obtained from the textxpert^®^ III software (Zwick Roell, Ulm, Germany). Figure 6 shows the fabricated scaffold samples’ representative mechanical properties (tensile and compressive modulus). It was seen that the elastic modulus of specimens was increased after surface modification, yet no statistical significance was found between groups. Statistically, the tensile modulus of PLA was 126.9 MPa, while the surface-modified group (PLA@PDA, PLA@PDA-HA3, and PLA@PDA-HA7) increased slightly to 148.1, 160.7, and 168.1 MPa respectively. On the other hand, the compressive modulus of scaffolds was significantly enhanced after immobilizing HA nanoparticles for 7 days. In detail, the compressive modulus of the PLA scaffold was 30.7 MPa, and it increased slightly to 32.4 MPa after introducing PDA to the surface. After surface functionalization with nHA for 3 days, the compressive modulus reached 34.2 MPa. More importantly, the compressive modulus of PLA@PDA-HA7 further increased to 39.2 MPa, significantly higher than the other three groups. However, it should be noted that no significant difference was recorded between PLA, PLA@PDA, and PLA@PDA-HA3 groups.

### Thermal gravimetric analysis (TGA)

**FIGURE 7:**
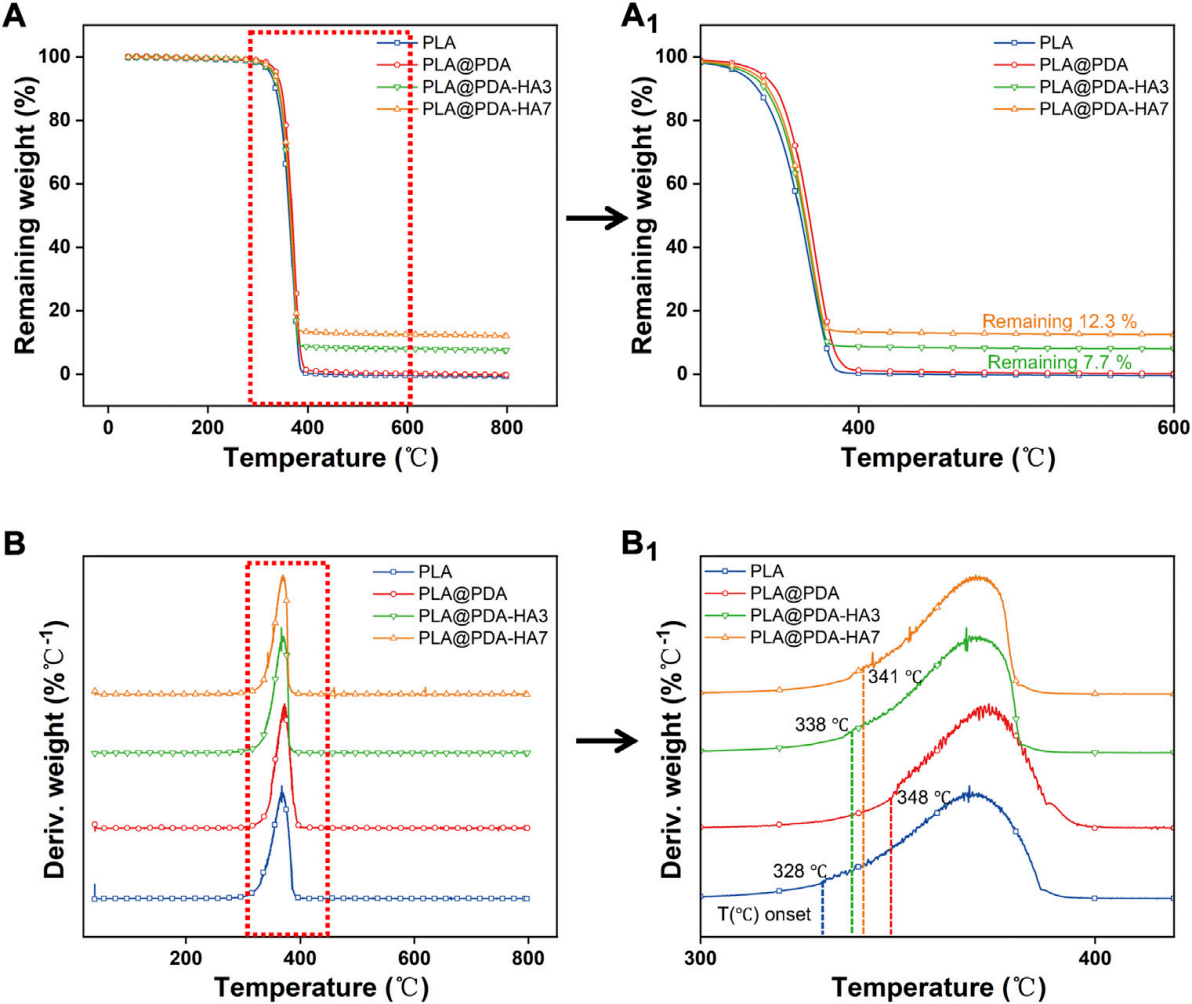
Thermal gravimetric curves (TGA) **(A)** and derivative thermal gravimetric curves (DTG) **(B)** of PLA, PLA@PDA, PLA@PDA-HA3, and PLA@PDA-HA7 scaffolds. Close-up views of TGA **(a**_**1**_ **)** and DTG **(b**_**1**_ **)** curves.

The thermal decomposition test was carried out to quantitatively determine the amount of HA nanoparticles deposited on the surface of PLA scaffolds. Figure 7 represents the TGA and DTG curves of different samples. According to Figure 7A, all samples showed a one-step thermolysis process at 328–380°C, which was evident by one peak from DTG curves (Figure 7B). The thermal degradation onset temperature for the PLA, PLA@PDA, PLA@PDA-HA3, and PLA@PDA-HA7 specimens were 328°C, 348°C, 338°C, and 341°C, respectively. For PLA and PLA@PDA specimens, no solid inorganic residues were found after the thermal decomposition, indicating that the tested samples were composed of pure organic components. After surface modification with nHA for 3 and 7 days, the mass ratio of 7.7 % and 12.3% inorganic residues was reported, corresponding to the amount of coated nHA on PLA@PDA-HA3, PLA@PDA-HA7 scaffolds, respectively.

### *In-vitro* degradation behavior of scaffolds

**FIGURE 8:**
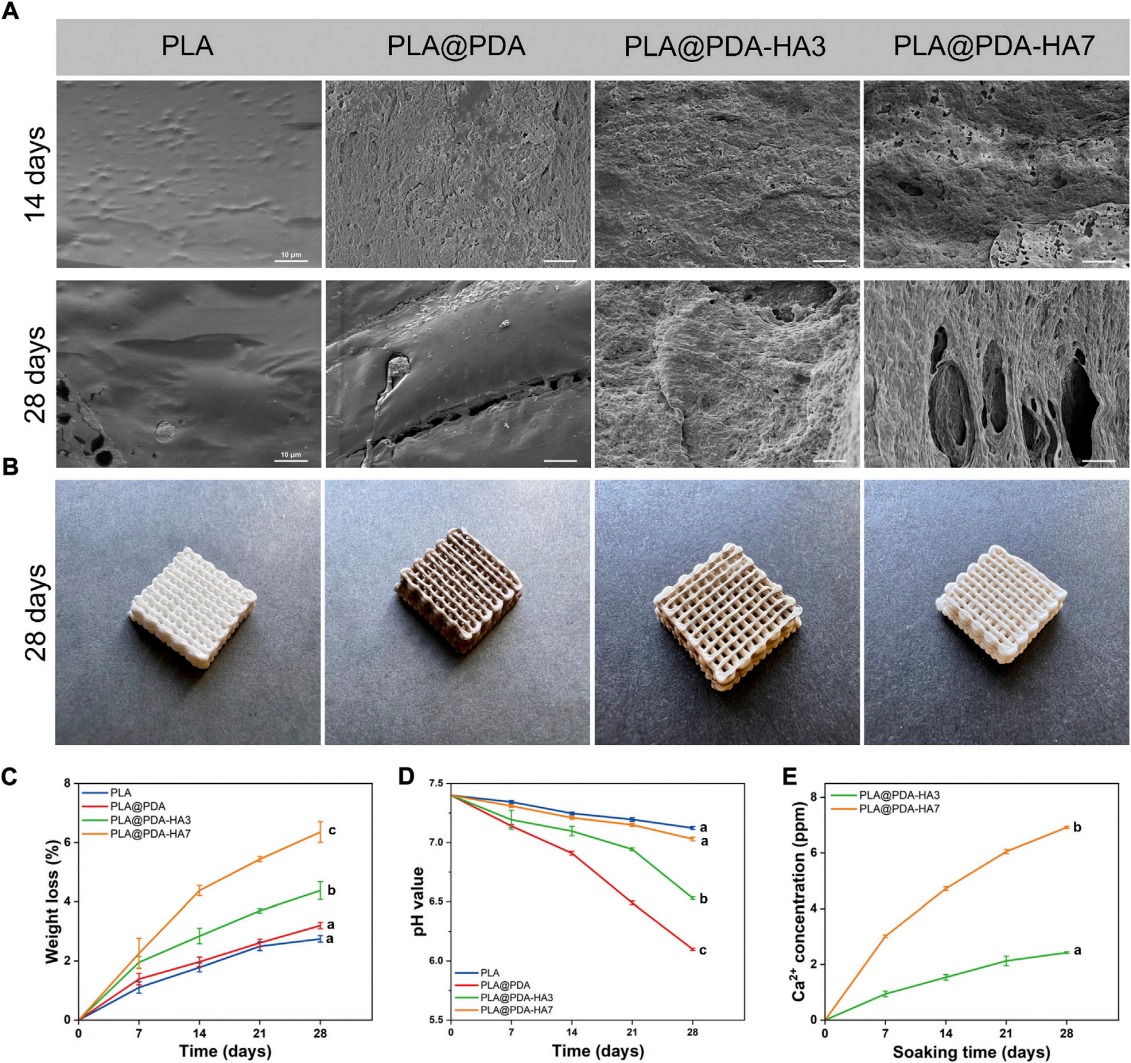
*In-vitro* degradation behavior of PLA, PLA@PDA, PLA@PDA-HA3, and PLA@PDA-HA7 scaffolds after immersing in PBS for 28 days. **(A)** SEM images of surface morphology of each scaffold after soaking in PBS for 14 and 28 days; **(B)** Representative optical photographs of each scaffold after 28 days of immersing; **(C)** Weight loss ratio of each scaffold for 28 days of degradation; **(D)** pH variation of immersing solution for 28 days; **(E)** 28 days’ Ca^2+^ release profile of PLA@PDA-HA3 and PLA@PDA-HA7 scaffolds.

The specimens were immersed in PBS solution for 28 days for *in-vitro* degradation analysis. According to Figure 8B, PLA@PDA, PLA@PDA-HA3, and PLA@PDA-HA7 scaffolds lightened in color after being soaked in PBS for 28 days, and all scaffolds showed no noticeable changes in their macrostructure, suggesting that the scaffolds were stable during 28 days of the degradation process. Figure 8A depicts the microstructure changes of different scaffolds on days 14 and 28. It can be seen that after 14 days of soaking in PBS, the microstructure of the PLA scaffold remained unchanged, exhibiting a relatively smooth and continuous surface with some tiny protuberances. In PLA@PDA scaffolds, visible eroded surfaces with tiny pits were observed after 14 days. After surface modification with nHA, degradation signs were visible. For the PLA@PDA-HA3 scaffold sample, an irregular surface with considerably large-sized pits was evenly distributed, and this trend became more evident in the PLA@PDA-HA7 scaffolds. After 28 days of soaking, the surfaces of PLA scaffolds were relatively intact, and only a few small-sized pits were formed.

Similarly, shallow cracks and minor holes were generated on the surface of PLA@PDA scaffolds. Interestingly, in contrast to the PLA and PLA@PDA scaffolds, nHA modification accelerated the degradation process for continuous immersion after 14 days. In the case of PLA@PDA-HA3, large-sized cracks and holes were easily observed. More importantly, PLA@PDA-HA7 exhibits large fractures that connect the surface to the inner side, indicating the highest degradation rate of all scaffolds after 28 days of immersion.

Accordingly, weight changes of scaffolds were recorded along with the degradation test. As depicted in Figure 8C, all scaffolds exhibited a continuous weight loss during 28 days of immersion. In detail, PLA showed the lowest mass loss, reaching 2.7% at 14 and 28 days, respectively. While PLA@PDA, PLA@PDA-HA3, and PLA@PDA-HA7 scaffold samples, exhibited a gradual increase in mass loss, reaching 3.2%, 4.4%, and 6.4% at 28 days, respectively. These results were in accordance with their degradation behavior.

Moreover, the pH variations of PBS solution for 28 days immersion period were recorded to understand the degradation behavior of all scaffolds. According to Figure 8D, the pH of all sample solutions gradually lowered with the extension of soaking time, indicating the formation of acidic by-products during the degradation process of PLA scaffolds. Specifically, the pH value for the PLA group tended to be more stable than the other three groups, reducing slightly to 7.1 during 28 days of immersion. On the other hand, the PLA@PDA specimen exhibited the most significant reduction in pH value, decreasing from 7.4 to 6.1 after 28 days of degradation. Interestingly, with the introduction of nHA to the surface, less reduction in pH value was observed, and such a tendency became clearer when nHA coating time was extended to 7 days. The pH value for PLA@PDA-HA3 decreased from 7.4 to 6.5, whereas the pH for PLA@PDA-HA7 was 7.0 at the end of the 28th day of incubation.

The calcium ion release properties of PLA@PDA-HA3 and PLA@PDA-HA7 scaffold samples were also measured at the various soaking period (Figure 8E). As expected, the PLA@PDA-HA7 exhibits the highest amount of Ca^2+^ releasing profile, almost three times that of PLA@PDA-HA3 samples. In detail, at the end of 7 days, the Ca^2+^ concentration for the PLA@PDA-HA7 sample reached 3.01 ppm, followed by 4.72, 6.05, and 6.92 ppm for 14, 21, and 28 days respectively. For the PLA@PDA-HA3 sample, the calcium ion concentration was 0.94, 1.53, 2.13, and 2.43 ppm at 7, 14, 21, and 27 days.

## Discussion

The present study aimed to comprehensively understand the physicochemical properties of surface bioactivated osteogenic PLA scaffolds fabricated using AM technology. The first hypothesis that the nHA and PDA layer would increase its hydrophilicity was proved by water uptake analysis and WCA measurement. The second hypothesis that the nHA and PDA coating would stimulate *in-vitro* hydrolysis of PLA scaffold was testified by SEM, mass loss study. Finally, the third hypothesis that the surface modification of the scaffolds would not lead to compromised mechanical strength was demonstrated by tensile and compressive strength tests.

In the last decade, PLA-based scaffolds produced by 3D printing technology had widespread uses in bone tissue engineering owing to their good biocompatibility, biodegradability, and printability (Kao et al., 2015; Hassanajili et al., 2019; Yao et al., 2020). However, the major drawbacks of PLA, such as hydrophobicity, slow degradation behavior, and lack of abundant cell-recognition functional group, limit further clinical application (Chen et al., 2021a). Until recently, researchers have proposed various approaches to optimize the osteogenic properties of the PLA to promote adequate cell interactions with its surface (Feng et al., 2018; Shuai et al., 2021). One of the promising approaches was integrating bioactive molecules and/or ceramics with PLA at the initial stage to optimize filaments for subsequent 3D printing. Generally, solvent-evaporation and mechanical-milling methods are typical approaches to fabricating PLA composites (Schliephake et al., 2015; Gayer et al., 2019). However, these approaches may result in undesirable outcomes, such as remaining toxic organic solvents and non-homogeneous composites with unpredictable biological performances (Gayer et al., 2019; Govindrao et al., 2019; Yu et al., 2019; Brounstein et al., 2021).

In the present study, HA nanoparticles were successfully introduced to the surface of 3D printed PLA scaffolds by organic solvent-free polydopamine coating. The present work studied the macrostructure and microstructure of 3D printed PLA scaffolds modified with PDA and nHA (Figure 2). After surface coating with PDA, the PLA scaffold’s color darkened significantly, ascribed to the formation of eumelanin on the surface. Similar color changes were also detected in PLA@PDA-HA3 and PLA@PDA-HA7 groups. Moreover, this result was further supported by the EDX, XRD, FTIR-ATR, and TGA tests. Specifically, as mentioned above, regarding the XRD result, the PLA@PDA-HA3 did not show the typical diffraction peaks of nHA particles, which is most probably ascribed to the low content of nHA. To meet the XRD test requirements, the PLA samples were printed in the shape of cylinders (D: 10 mm; H: 1 mm) before coating with PDA and nHA according to previously reported literature (Kim et al., 2021). Due to the small-size and porous-free structure of the specimens, a relatively lower amount of nHA was immobilized onto the surface after 3 days of immersion, which was under the lower limit of detection of XRD analysis. Regarding the microstructure of PLA scaffolds, SEM tests confirmed that the interconnected pore size was ∼400 μm, which was in accordance with the previously described optimal pore size for bone regeneration (200–500 μm) (Thavornyutikarn et al., 2014). Additionally, controlled porosity (36% ± 0.005 (*p* < 0.05)), which was within the range of cancellous bone, of the 3D-printed PLA scaffolds was detected (Bose et al., 2012).

Another major limitation of the 3D-printed PLA scaffold is its hydrophobic properties, which were not conducive for osteoblasts to promote bone formation (Mohsenimehr et al., 2020). Indeed, the hydrophilicity of biomaterials is a pivotal factor affecting initial cell adhesion (Boyan et al., 2017). The present study achieved significantly improved surface hydrophilicity by PDA coating (WCA from 92.7° to 52.5°). Previous studies have pointed out that the PDA contains many hydrophilic functional groups, such as amino groups, which enhance surface wettability by promoting hydrogen bonding (Li et al., 2019). More interestingly, after immobilizing nHA on the PLA@PDA surface, the water droplet was absorbed instantly after it touched the surface, demonstrating that the nHA coating could further turn its surface into a superhydrophilic surface (WCA = 0°). It should be noted that apart from the abundant hydroxyl groups from HA that enhance the surface hydrophilicity to a further extent, nanosized HA particles with increased specific areas exposed to the surface were also reportedly contributing to the results above (Crisan et al., 2015). As a result, by achieving a hydrophilic surface, the nHA-coated PLA scaffolds are expected to be covered by serum more rapidly to provide bioactive molecular factors that can further stimulate cells to proliferate and osteogenic differentiate at an initial stage.

The micro-topography of the scaffold surface is another decisive factor for their osteogenic behavior since it is the first site for surrounding cells and tissues in contact (Cai et al., 2020). There have been controversial reports regarding the correlation between surface roughness and osteogenic behavior. For instance, literature has demonstrated that the optimal osteogenic properties were realized on micro-scale surface roughness (Sa = 3–4 μm) (Costa et al., 2013). In contrast, Wong et al. reported that a relatively smooth surface roughness (Sa = 0.22 μm) provided a good platform for osteoblastic MG63 cell attachment and proliferation (Wong et al., 2020). However, most preclinical studies focused on elucidating the mechanism of how surface roughness directly affects (pre-) osteoblastic cell behavior solely. Apart from osteoblasts, osteoclasts have a remarkable impact on bone formation since they reportedly initiate the bone remodeling process earlier than osteoblastic cells (Orsini et al., 2012). A recent study by Zhang et al. revealed the relationship between surface roughness and bone formation by uncovering the mechanism of cross-talk between osteoblasts and osteoclasts (Zhang et al., 2018). The results showed that surface roughness is an essential factor for osteogenesis and osteoclastogenesis, which positively affects the osteogenic differentiation of osteoblastic cells far more obviously than osteoblast itself. Accordingly, although osteoblastic cell differentiation was optimized on micro rough surfaces (ca. Sa = 3–4 μm), the authors suggested smooth surfaces were more suitable for bone formation (Zhang et al., 2018). In the present study, the surface roughness of PLA scaffolds gradually increased with the extension of nHA coating time. As a result, the Sa of PLA@PDA-HA7 reached 0.40 μm, however, it could still be regarded as a relatively smooth surface (<1 μm) compared to micro-scale rough surfaces. Further studies are needed to clarify the desired surface roughness for different cell and tissue types to achieve the desired outcomes.

The mechanical properties of 3D printed scaffolds are important factors determining their *in vivo* performance for bone regeneration in the long term (Prasadh and Wong, 2018). They typically undergo different mechanical stress such as compression and tension from surrounding bone tissues, affecting their stability and osseointegration properties (Little et al., 2011). Therefore, the mechanical properties of scaffolds should be tailored to match the native bone to be repaired (Little et al., 2011). After surface modification, the mechanical behaviors of scaffolds should be characterized before implantation to avoid possible implant failure. In the present study, the tensile and compressive tests were carried out for all scaffolds to determine the changes in their mechanical properties before and after surface modification. Regarding tensile tests, although there was no statistical significance among all four groups, the tensile modulus increased slightly after surface modification and reached its highest level of 168 MPa in the PLA@PDA-HA7 group. On the other hand, the compressive strength of PLA@PDA-HA7 scaffolds was significantly higher than PLA and PLA@PDA groups; however, not statistically significant compared to the PLA@PDA-HA3 group. Taking all results together, it can be concluded that the PDA and nHA coating on the surface of PLA scaffolds would grant them enhanced resistance and were less prone to deformation. These results could be attributed to two reasons. First, the nHA and PDA coating acts as an adhesive transition layer, resulting in a favorable stress buffer between the coating layer and scaffold surface (Guan et al., 2018). Second, the uniformly dispersed nanosized HA particles with a high modulus act as a buffer zone that consumes more fracture energy (Meng et al., 2019).

**FIGURE 9:**
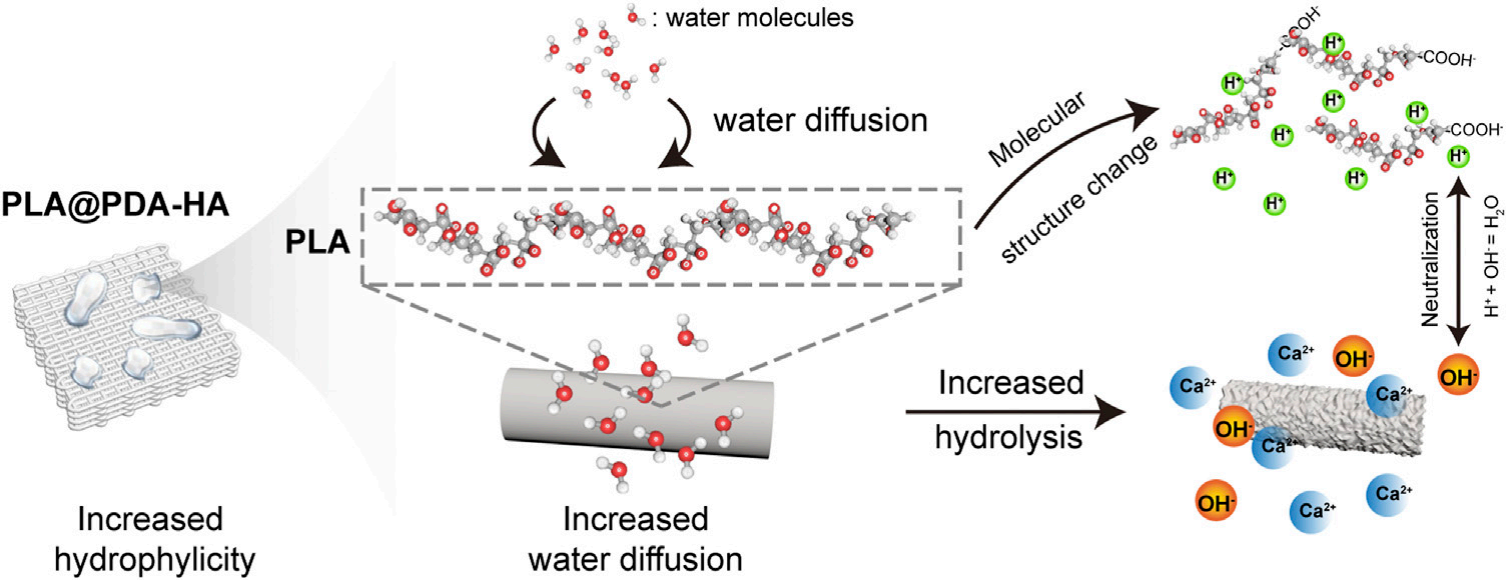
Schematic illustration of hydrolysis process of PLA@PDA-HA scaffolds. The increased surface wettability of nHA-modified PLA scaffolds enhances water diffusion, by which PLA chains are “attacked” by a more significant number of water molecules. As a result, an accelerated hydrolysis process, namely the cleavage of the ester bond, took place, and in turn, a local acidic environment was formed due to the exposure of the carboxyl group. The hydroxyl group from nHA and PDA neutralized the local acids greatly.

Generally, biodegradation of bone grafting substitutes is achieved during the repair of bone defects, which requires a consistent rate between new bone formation and artificial implant scaffold degradation (Bose et al., 2012). Although 3D-printed PLA-based scaffolds are biodegradable, the degradation process is deemed to be too slow (2–3 years) to match the rate of *in vivo* new bone formation (8–12 weeks) (Feng et al., 2021). Theoretically, in a physiological environment, the PLA-based implant scaffold is mainly degraded by the action of water molecules (Feng et al., 2021). In the present study, enhanced biodegradable properties were achieved by introducing HA nanoparticles to the surface of 3D printed PLA scaffolds, as evidenced by Figure 7. As discussed above, the primary mechanism of PLA scaffold degradation is hydrolysis. Thus, it was speculated that the improved hydrophilicity of the PLA scaffold surface increased water intake and initiated the corrosion of the surface, which in turn promoted the immersion of water molecules. Apart from increased hydrophilicity of scaffold surface, nHA coating is another important factor that accelerates the degradation. According to previous literature (Yuan et al., 2002), the PLA-based scaffolds degrade much faster in an alkaline environment than in an acidic solution. As illustrated in Figure 9, Ca^2+^ released from the PLA@PDA-HA3 and PLA@PDA-7 scaffold formed hydroxide, thereby creating a local micro-alkaline environment, further accelerating the degradation of PLA scaffolds. The results suggested that the PLA@PDA-HA7 possesses the fastest degradation rate among all scaffolds, mainly due to its superior hydrophilicity and nHA coating. Notably, after 28 days of immersion, the mass loss ratio of PLA@PDA-HA3 and PLA reached 4.4 % and 6.4%, respectively; however, the macrostructure remains unchanged, indicating the degradation was mainly confined to the surface area of the scaffold and did not affect the overall stability during the initial degradation process.

Meanwhile, the pH variation during 28 days of immersion time was recorded. Proverbially, the acidic environments produced during the biodegradation of PLA scaffolds might cause a local immune response, which is a detrimental factor for bone regeneration (Feng et al., 2021; Shuai et al., 2021). The results from the present immersion test revealed that the nHA coating layer greatly neutralized the acidic by-products produced during the degradation process of the PLA scaffold, and such trends became more evident with the increasing amount of HA nanoparticles on the surface. A plausible explanation for this result is that the nHA possesses weak alkalinity, and the hydroxyl group of nHA acts as a buffer to suppress the acidic environment (Wang et al., 2021). In contrast, the PDA coating layer seems to “accelerate” the acidic formation, as the pH decreased to 6.1 after 28 days of immersion. On the one hand, as discussed above, the increased hydrophilicity of the PLA scaffolds achieved by the PDA coating layer would enhance their hydrolysis, which promotes the formation of acidic degradation products of the PLA. On the other hand, the acidic functional group of PDA, namely, the catechol group, is exposed while the degradation progress, resulting in a decrease in the local pH value (Chen et al., 2021b).

Moreover, according to the calcium ion release profile (Figure 8E), both nHA-modified PLA scaffolds showed a “fast followed by slow” trend during 28 days of immersion. At the end of 28 days of soaking, the accumulated Ca^2+^ concentration released by PLA@PDA-HA3 and PLA@PDA-HA7 reached 2.43 ppm (0.06 mM) and 6.92 ppm (0.173 mM), respectively. Previous studies have demonstrated the correlation between extracellular Ca^2+^ concentration and osteogenic behavior. For instance, upregulated alkaline phosphatase (ALP) and osteocalcin (OCN) expression of MC3T3-E1 cells was detected when local Ca^2+^ concentration reached 8 ppm (Park et al., 2010), with similar Ca^2+^ concentration reported in the present study. More importantly, Park et al*.* previously reported that the scaffold’s ability to increase local Ca^2+^ concentration is more critical than its surface’s roughness and hydrophilicity, promoting osteogenic differentiation. Other studies have also revealed that the surface functionalization with bioactive molecules such as Ca^2+^ could greatly enhance early cellular response and subsequent osteogenic behavior such as extracellular mineralization (Kim et al., 2016). Thus, considering all these results, the nHA coating layer could provide surrounding cells and tissues with a conducive environment for bone formation by neutralizing the pH of the local environment and providing sufficient Ca^2+^ to optimize their osteoconductive and osteoinductive properties.

Generally, additively manufactured 3D scaffolds for bone tissue engineering have a wide range of advantages over conventional fabrication methods in terms of cost, time effectiveness, and flexibility in designing patient-specific implants (Zhang et al., 2019). Nevertheless, limited material resources for 3D printing make it hard to pick specific materials for the desired application. On the other hand, traditionally available biomaterials are not feasible for 3D printing, while the best performing AM materials, in terms of printability and accuracy, are often not osteoconductive and hard to biodegrade (Zhang et al., 2019; Qu, 2020). In this study, we provide a facile approach by combining the traditionally available techniques of surface modification with the cutting-edge technology of AM to better engineer patient-specific bone regenerative scaffolds. The findings presented in this study, physiochemical properties of biomimicry produced PLA@PDA-HA scaffolds, would grant effective application both in bone regenerative medicine and biomedical engineering. Furthermore, considering the relatively low costs of PLA and nHA materials, the present study provided, for the first time, a comprehensive understanding and evaluation of the effects of nHA functionalized 3D printed PLA scaffolds, from their design, fabrication, and post-process to mechanical behavior, surface physicochemical properties, and biodegradability. These findings provide insights into how AM methods could improve the application potential in bone tissue engineering. The authors believe that such a multidisciplinary technology of post-processing additively manufactured scaffolds would foresee a hopeful paradigm shift in the field of regenerative medicine. Further studies, both *in-vitro* and *in-vivo* biological performance, should be carried out in the near future to determine osteogenic efficacy before clinical application.

## Conclusion

In the present study, PDA mediated coating method was applied to successfully immobilize HA nanoparticles onto the surface of PLA scaffolds fabricated by material extrusion-based 3D printing technology. Such a facile, mussel-inspired post-processing method enables nHA to be highly loaded on the PLA scaffold surface after 3 and 7 days of immersion, resulting in a 7.7 % and 12.3% mass ratio, respectively. The surface functionalization with PDA and nHA effectively increased the hydrophilicity of PLA scaffolds, which greatly enhanced the surface hydrolysis process of the PLA scaffolds. Furthermore, mechanical behavior and surface macrostructures were not compromised thanks to the post-modification method used in this research. Instead, significantly enhanced compressive modulus and surface roughness was detected in the PLA@PDA-HA7 group. With these encouraging results, nHA functionalized PLA scaffolds have great potential in bone tissue engineering.

## Data Availability

The original contributions presented in the study are included in the article/Supplementary Material, further inquiries can be directed to the corresponding authors.
