# Supplementary material for: Biomimetic, mussel-inspired surface modification of 3D-printed biodegradable polylactic acid scaffolds with nano-hydroxyapatite for bone tissue engineering
Source: Front Bioeng Biotechnol. 2022 Sep 8;10:989729. doi: 10.3389/fbioe.2022.989729 (PMC9493000; doi:10.3389/fbioe.2022.989729)
Supplement: Supplementary file 1 [file DataSheet1.docx]

Supplementary Material

## Supplementary Figures


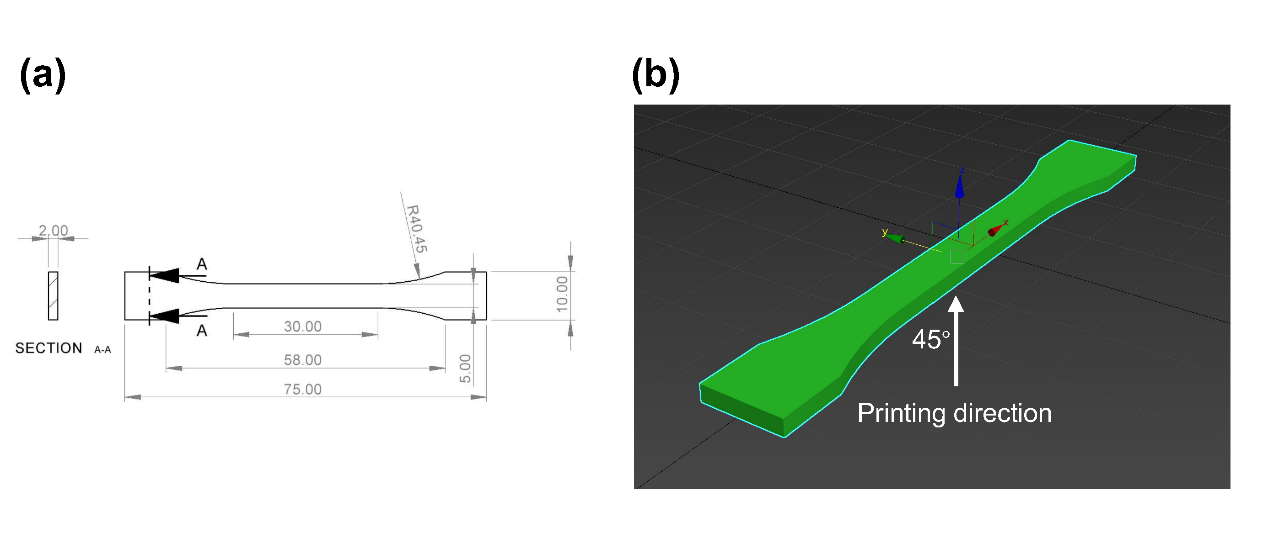


**Supplementary Figure S1.** Graphical demonstration of the parameters (a) and printing direction (b) of the tensile specimen according to ISO 527-1 (2012) standard.


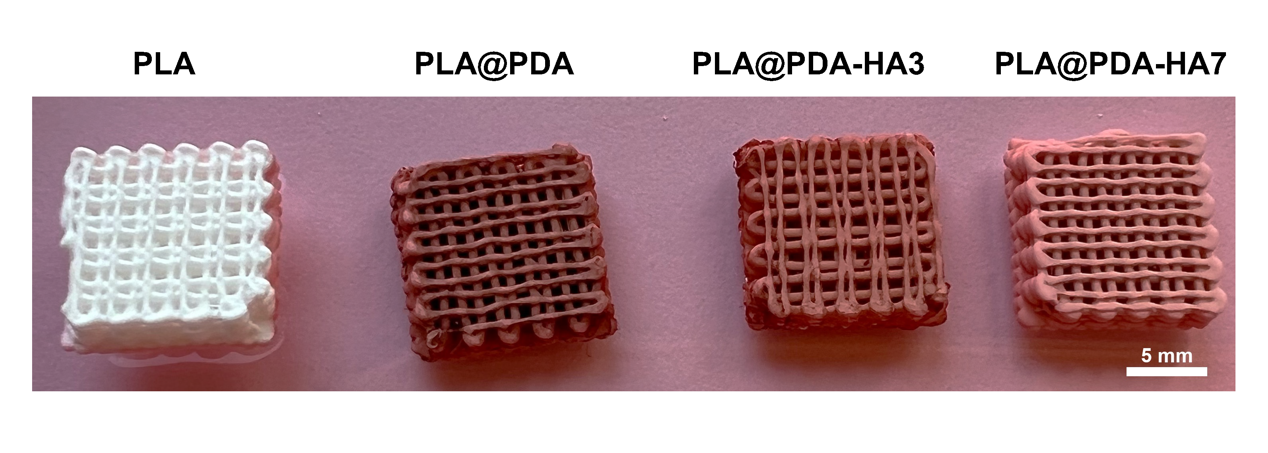


**Supplementary Figure S2.** Digital photographs illustrating water-uptake analysis. The PLA scaffold without any modification showed poor wettability, as it floated on the PBS solution. In contrast, surface-modified specimens (PLA@PDA, PLA@PDA-HA3, and PLA@PDA-HA7 sank to the bottom of the PBS solution, showing enhanced hydrophilicity.

**Supplementary Table S1: Porosities of 3D-printed PLA scaffolds**

|  | **ρ _scaffolds_ (g/cm^3^)** | **ρ _pla_ (g/cm^3^)*** | ***Porosity from Eq. (1)*** | | ***Cancellous bone (%) ***** |
| --- | --- | --- | --- | --- | --- |
| ***PLA scaffolds (n=6)*** | 0.79 ± 0.006 | 1.25 | | 36 ± 0.005 | 30 — 90 |

* Density values for PLA were reported by Van et al. (Van der Walt et al., 2019)

** Porosity values for cancellous bone were reported by Bose et al. (Bose et al., 2012)

## References

BOSE, S., ROY, M. & BANDYOPADHYAY, A. 2012. Recent advances in bone tissue engineering scaffolds. *Trends in biotechnology,* 30**,** 546-554.

VAN DER WALT, M., CRABTREE, T. & ALBANTOW, C. 2019. PLA as a suitable 3D printing thermoplastic for use in external beam radiotherapy. *Australasian physical & engineering sciences in medicine,* 42**,** 1165-1176.
